# Pathway-level analysis suggests a maintenance-oriented transcriptional response of the ammonia-oxidizing bacterium *Nitrosospira* sp. NT to acidic pH

**DOI:** 10.3389/fmicb.2026.1957381

**Published:** 2026-10-08

**Authors:** Yuxiang Song, Zhongjun Jia

**Affiliations:** 1 State Key Laboratory of Soil and Sustainable Agriculture, Institute of Soil Science, Chinese Academy of Sciences, Nanjing, Jiangsu, China; 2 University of Chinese Academy of Sciences, Beijing, China; 3 State Key Laboratory of Black Soils Conservation and Utilization, Northeast Institute of Geography and Agroecology, Chinese Academy of Sciences, Changchun, Jilin, China

**Keywords:** acidic pH, ammonia oxidation, cellular maintenance, genome-resolved metatranscriptomics, metabolic reorganization, nitrification

## Abstract

Acidic environments impose energetic constraints on ammonia-oxidizing bacteria (AOB), although some acid-tolerant lineages remain capable of ammonia oxidation at low pH. The transcriptional responses of acid-tolerant AOB to low-pH conditions remain poorly resolved. Here, we characterized the pathway-level response of *Nitrosospira* sp. NT by integrating a reconstructed metagenome-assembled genome with comparative metatranscriptomic data from cultures incubated for 24 h at pH 5.0 or pH 8.0. Transcript abundance was not uniformly reduced among the metabolic pathways examined at pH 5.0. Genes associated with the Calvin–Benson–Bassham cycle, the oxidative tricarboxylic acid cycle, nucleotide and deoxyribonucleotide biosynthesis, glycogen metabolism, and septal peptidoglycan synthesis generally showed lower transcript abundance, indicating reduced expression of pathways linked to biomass production and cell proliferation. By contrast, transcripts associated with selected oxidative pentose phosphate pathway enzymes, cell-envelope homeostasis, membrane-protein quality control, c-di-GMP-associated surface functions, stored-iron mobilization, and c-type cytochrome maturation showed comparable or higher transcript abundance. Iron-dependent cofactor pathways also responded differently: transcripts related to bacterioferritin-associated iron mobilization and Ccm-dependent cytochrome c maturation were higher, whereas those involved in heme a/o synthesis and SUF-mediated Fe–S cluster assembly were lower. Collectively, these patterns support a maintenance-oriented transcriptional model in which transcripts associated with cellular integrity, redox support, envelope homeostasis, and intracellular iron reutilization were relatively more abundant than those associated with biomass accumulation at pH 5.0. This study provides a pathway-level framework for understanding the transcriptional response of acid-tolerant AOB to acidic conditions.

## 1 Introduction

Nitrification is a central process in the global nitrogen cycle, and ammonia oxidation is its initial and often rate-limiting step (Kuypers et al., 2018). This process is mediated by ammonia-oxidizing bacteria (AOB), ammonia-oxidizing archaea (AOA), and complete ammonia oxidizers (comammox) (Konneke et al., 2005; Martens-Habbena et al., 2009; van Kessel et al., 2015). Many AOA lineages are adapted to low ammonia availability and low-pH conditions and are therefore considered important drivers of nitrification in acidic environments (Martens-Habbena et al., 2009; Gubry-Rangin et al., 2011; Lehtovirta-Morley et al., 2011; Zhang et al., 2012). Nevertheless, accumulating evidence shows that AOB can also remain active and contribute substantially to nitrification at low pH (Huang et al., 2018; Zhang et al., 2019). Acid-tolerant AOB may grow more slowly or exhibit lower activity under strongly acidic conditions than at their pH optima, demonstrating that acid tolerance does not imply optimal growth or metabolic performance (Hayatsu et al., 2017; Picone et al., 2021). As chemolithoautotrophs, AOB derive energy and reducing power from ammonia oxidation. Because this metabolism provides a relatively low energetic yield for anabolism, available energy must support competing demands, including CO_2_ fixation, biosynthesis, cellular maintenance, and stress responses (Chain et al., 2003; Mellbye et al., 2016; Gonzalez-Cabaleiro et al., 2019). Acid stress adds further costs associated with intracellular pH regulation, membrane maintenance, redox homeostasis, and protein quality control (Chang and Cronan, 1999; Mols et al., 2010; Senouci-Rezkallah et al., 2015; Zhang et al., 2016). How acid-tolerant AOB adjust gene expression across these competing demands remains poorly understood.

Acidic conditions can constrain AOB activity through several non-mutually exclusive mechanisms (Allison and Prosser, 1993; Jiang and Bakken, 1999). NH_3_ is generally considered the direct substrate of ammonia monooxygenase; as pH declines, however, the proportion of NH_3_ relative to total ammoniacal nitrogen decreases, thereby limiting substrate availability (Suzuki et al., 1974). Cells must also expend additional energy to maintain intracellular pH, membrane potential, and ionic homeostasis (Sun et al., 2012; Sanchez-Andrea et al., 2022). Moreover, low pH can affect protein stability and metal availability (Gajiwala and Burley, 2000; Dopson et al., 2014). Bacterial responses to acid stress can involve ion transport, membrane remodeling, protein quality control, and other mechanisms that contribute to intracellular homeostasis (Lund et al., 2020). Members of the genus *Nitrosospira* have been reported from soils and freshwater sediments spanning a range of pH conditions (Shen et al., 2012; Urakawa et al., 2015). DNA-based stable isotope probing has further identified *Nitrosospira* as active, and in some cases dominant, contributors to nitrification in acidic environments (Huang et al., 2018; Zhang et al., 2019). Genomes of representative *Nitrosospira* strains encode ammonia oxidation, respiratory electron transport, inorganic carbon fixation through the Calvin–Benson–Bassham (CBB) cycle, and partial nitrogen-oxide metabolism, including nitrite and nitric-oxide reduction (Norton et al., 2008; Rice et al., 2016). However, how acid-tolerant *Nitrosospira* coordinate these functions to maintain essential cellular functions under acidic conditions remains unclear.

A key unresolved question is not simply whether acidic conditions suppress growth-associated functions, but how acid-tolerant AOB selectively reorganize transcription among competing cellular processes while remaining metabolically viable at low pH. Ammonia oxidation supports relatively low biomass yields, and acid stress imposes additional energetic demands associated with intracellular homeostasis and cellular protection (Lehtovirta-Morley et al., 2016; Mellbye et al., 2016; Zorz et al., 2018; Gonzalez-Cabaleiro et al., 2019; Peterse et al., 2026). Under these constraints, pathways supporting carbon assimilation, nucleotide synthesis, DNA replication, and cell division may respond differently from functions involved in membrane and redox homeostasis, respiration, environmental sensing, and cofactor maintenance. Importantly, these maintenance-associated processes may themselves respond nonuniformly, making it necessary to resolve the response at the pathway and functional-module levels rather than infer a generalized stress response. Recent studies have begun to apply genome-resolved transcriptomics to investigate nitrifier responses across pH gradients (Li et al., 2025; Peterse et al., 2026; Zhang et al., 2026). However, coordinated divergence among growth-associated, maintenance-associated, and cofactor-related pathways has not been specifically characterized in acid-tolerant *Nitrosospira*. Resolving this organization would provide a pathway-level framework for understanding how bacterial ammonia oxidizers maintain essential cellular functions and potentially persist as active nitrifiers under acidic conditions.

In this study, we examined the transcriptional response of *Nitrosospira* sp. NT within an acid-tolerant ammonia-oxidizing enrichment culture after 24 h of incubation at pH 5.0 or pH 8.0. Gene-level expression profiles were mapped onto key metabolic and cellular processes, including the CBB cycle and central carbon metabolism, nucleotide and deoxyribonucleotide biosynthesis, cell-envelope remodeling, c-di-GMP-associated surface functions, septal peptidoglycan synthesis, iron acquisition, heme and Fe–S metabolism, and oxidative phosphorylation. We investigated whether these cellular functions respond uniformly to acidic pH or display distinct pathway-specific transcriptional patterns. Specifically, we assessed whether pathways associated with biomass production and cell proliferation were reduced relative to cellular-maintenance functions, and whether individual maintenance and cofactor-related modules showed distinct transcriptional responses. This pathway-level analysis therefore aimed to resolve which cellular functions are selectively reduced, preserved, or enhanced under acidic conditions.

## 2 Materials and methods

### 2.1 Enrichment culture and experimental design

An acid-tolerant ammonia-oxidizing enrichment culture containing *Nitrosospira* sp. NT as the predominant ammonia oxidizer was used in this study. Despite repeated attempts using conventional serial-transfer and enrichment procedures for approximately 1 year, *Nitrosospira* sp. NT has not yet been obtained in pure culture. Therefore, a genome-resolved metagenomic and metatranscriptomic approach was used to characterize its transcriptional response within the enrichment culture. The culture was originally established from soil collected from a maize field at the Lishu Conservation Tillage Research and Development Station, Institute of Applied Ecology, Chinese Academy of Sciences, in Jilin Province, Northeast China (43°19′ N, 124°14′ E). It was maintained aerobically in AOB medium, with ammonium supplied as the sole energy source.

The enrichment was routinely maintained at pH 6.0 as a moderately acidic selective condition; this maintenance pH was not intended to represent the physiological optimum of the enrichment culture. Before the pH-shift experiment, the enrichment culture was grown at pH 6.0 to the exponential phase. Exponentially growing cultures were divided between two treatments: pH 5.0, representing a strongly acidic condition near the lower boundary of detectable nitrification activity of the enrichment culture, and pH 8.0, representing the pH range associated with maximal nitrification activity. Culture pH was adjusted with sterile 1 M HCl or 1 M NaOH, as appropriate, and the cultures were incubated for 24 h under otherwise identical conditions. The pH was monitored regularly and readjusted as necessary to maintain the target values. Each treatment included three independent biological replicates (*n* = 3), resulting in six metatranscriptomic samples in total. Samples were collected after 24 h for metatranscriptomic analysis.

### 2.2 Genome reconstruction and functional annotation of *Nitrosospira* sp. NT

Because *Nitrosospira* sp. NT was maintained in an enrichment culture rather than as a pure culture, metagenomic sequencing was used to reconstruct its genome and generate an organism-specific reference for subsequent metatranscriptomic analysis. Genomic DNA was extracted from the exponentially growing parent enrichment culture before the pH treatments were established. Metagenomic sequencing of the exponentially growing parent enrichment culture was performed using three independent biological replicates before the pH treatments were established. Cells from each replicate were harvested, and genomic DNA was extracted using the Fast DNA SPIN Kit (MP Biomedicals, Cleveland, OH, USA) according to the manufacturer’s instructions. DNA quality and concentration were assessed using a NanoDrop 2000 spectrophotometer (Thermo Scientific, Wilmington, DE, USA) and 1% agarose gel electrophoresis. Sequencing libraries were constructed using the TruSeq DNA Sample Preparation Kit (Illumina, San Diego, CA, USA) and sequenced using an Illumina HiSeq platform.

Raw sequencing reads were quality-filtered using fastp v0.23.4 (Chen et al., 2018). For taxonomic profiling of the parent enrichment culture, quality-filtered paired-end reads were classified using Kraken2 v2.0.7-beta (Wood et al., 2019) in paired-end mode with the --use-names option against a locally installed RefSeq database. Clean reads were assembled *de novo* using MEGAHIT v1.2.9 (Li et al., 2015). The assembled contigs were grouped into metagenome-assembled genomes (MAGs) using MaxBin v2.2.7 (Wu et al., 2016). Genome completeness and contamination were evaluated using CheckM2 v1.1.0 (Chklovski et al., 2023). Taxonomic classification of MAGs was performed using GTDB-Tk v2.4.0 (Chaumeil et al., 2022). The MAG corresponding to *Nitrosospira* sp. NT was selected based on taxonomic assignment and the presence of genes associated with ammonia oxidation. Functional annotation was performed using KofamScan v1.3.0 against the KOfam/KEGG Orthology database (Aramaki et al., 2020). KO assignments were retained according to the KOfam profile-specific score criteria. The reconstructed genome of *Nitrosospira* sp. NT was classified as a high-quality MAG, with 99.0% completeness and 1.6% contamination. It comprised 3,352,347 bp, with a coding density of 86.1%, a GC content of 54.0%, and 3,189 predicted coding sequences, and was used as the reference for metatranscriptomic read mapping and downstream functional analyses.

To characterize the taxonomic composition of the parent enrichment culture, species-level taxonomic counts were obtained from the Kraken2 reports for the three biological replicates collected before establishment of the pH treatments. For each replicate, reads assigned to individual taxa were normalized to the total number of taxonomically assigned reads in that sample to calculate relative genomic representation. Major species-level taxa were visualized as stacked bar plots in Supplementary Figure S1A.

### 2.3 Metatranscriptomic sequencing and differential expression analysis

To characterize transcriptional responses to acidic pH, RNA samples were collected from the pH 5.0 and pH 8.0 treatments after 24 h of incubation. Cells from each biological replicate were collected by centrifugation, and total RNA was extracted using the RNeasy PowerSoil Total RNA Kit (Qiagen, Valencia, CA, USA) according to the manufacturer’s instructions. RNA quality was evaluated using a NanoDrop 2000 spectrophotometer, agarose gel electrophoresis, and an Agilent 5300 Fragment Analyzer (Agilent Technologies, Santa Clara, CA, USA). RNA libraries were constructed using the TruSeq RNA Sample Preparation Kit (Illumina, San Diego, CA, USA) and sequenced on an Illumina HiSeq platform.

Raw reads were filtered using fastp v0.23.4 (Chen et al., 2018). High-quality reads from each sample were quantified against the predicted coding sequences of the reconstructed *Nitrosospira* sp. NT MAG using Salmon v1.10.3 with the parameters -l A --validateMappings --seqBias --gcBias (Patro et al., 2017). Salmon-derived estimated counts were used for differential expression analysis, whereas transcripts per million (TPM) values were used only to summarize relative transcript abundance (Wagner et al., 2012).

Differentially expressed genes between the pH 5.0 and pH 8.0 treatments were identified using DESeq2 in R, with pH 8.0 as the reference condition. Genes with an absolute log_2_ fold change ≥1 and a Benjamini–Hochberg-adjusted *p* value < 0.05 were considered significantly differentially expressed. Positive log_2_ fold change values indicate higher expression at pH 5.0, whereas negative values indicate lower expression at pH 5.0. Throughout the manuscript, “significantly upregulated” and “significantly downregulated” refer to genes meeting both predefined differential-expression criteria, whereas “higher transcript abundance” and “lower transcript abundance” are used for directional differences that do not meet both predefined differential-expression criteria or for pathway-level summaries involving genes with mixed statistical significance. The resulting expression profiles were mapped onto selected KEGG pathways for pathway reconstruction and directional visualization. Gene-level expression data used for pathway reconstruction, including KO assignments, TPM values, log_2_ fold changes, adjusted *p* values, and DEG classifications, are provided in Supplementary Table S1.

To characterize the taxonomic representation of the complete metatranscriptomic libraries, quality-filtered paired-end reads from all six samples were classified using Kraken2 v2.0.7-beta in paired-end mode with the --use-names option. The same locally installed RefSeq database used for the metagenomic analysis was applied to ensure consistent taxonomic classification between the DNA- and RNA-based datasets. Species-level taxonomic counts were obtained from the Kraken2 reports and normalized to the total number of taxonomically assigned reads in each library to calculate relative transcript representation. Major transcriptionally represented taxa were visualized in Supplementary Figure S1B. Because taxon-specific read representation in metatranscriptomic libraries reflects both population abundance and transcriptional activity, these RNA-based profiles were used only to describe relative transcriptional representation and were not interpreted as direct estimates of population-level community composition or relative abundance.

Sample-level reproducibility and overall transcriptional variation of *Nitrosospira* sp. NT were evaluated using the CDS-level count matrix. CDSs with zero counts across all six libraries were excluded from these analyses. Counts were normalized using DESeq2 size factors and transformed as log_2_(normalized count+1). Pairwise Pearson correlation coefficients were calculated across the retained CDSs and visualized as a sample-correlation heatmap. Principal component analysis was performed on the same transformed count matrix after centering the variables, without additional variance scaling, to assess the major sources of variation among samples. The percentages of total variance explained by the first two principal components were calculated from the corresponding eigenvalues.

Genome-wide differential-expression patterns were visualized using an MA plot generated from the DESeq2 results. For each CDS, the DESeq2 baseMean was log_10_-transformed and plotted against the log_2_ fold change for pH 5.0 relative to pH 8.0. Genes with |log_2_ fold change| ≥ 1 and a Benjamini–Hochberg-adjusted *p* value < 0.05 were classified as significantly differentially expressed; genes not meeting both criteria were retained as non-DEGs for visualization. Genes with positive and negative log_2_ fold-change values meeting these criteria were classified as significantly upregulated and significantly downregulated, respectively.

## 3 Results

Metagenomic taxonomic profiling of the parent enrichment showed that a *Nitrosospira multiformis*-related population represented approximately 13.8% of the metagenomic taxonomic profile and was the most abundant species-level taxonomic bin (Supplementary Figure S1A). Other abundant members included *Dokdonella koreensis* (~12.8%), *Nitrobacter hamburgensis* (~10.1%), *Pseudomonas putida* (~5.1%), and *Nakamurella multipartita* (~4.1%). Major transcriptionally represented taxa, including *Nitrosospira*, *Pseudomonas*, *Nakamurella*, and *Chitinophaga*, were detected under both pH conditions, although their relative transcript representation differed between treatments (Supplementary Figure S1B). Because treatment-specific DNA-based community profiles were not obtained, these metatranscriptomic differences cannot be interpreted as evidence of changes in population-level community composition.

The transcriptomic profiles showed high within-treatment reproducibility, with pairwise Pearson correlations of 0.983–0.984 among the pH 5.0 replicates and 0.979–0.988 among the pH 8.0 replicates (Supplementary Figure S2A). PCA separated the two pH treatments primarily along PC1, which explained 76.8% of the total variance, although the pH 8.0 R2 sample showed some separation along PC2 (Supplementary Figure S2B). Genome-wide differential-expression analysis of *Nitrosospira* sp. NT identified 694 significantly differentially expressed genes at pH 5.0 relative to pH 8.0, including 438 significantly upregulated and 256 significantly downregulated genes (|log_2_ fold change| ≥ 1 and adjusted *p* < 0.05; Supplementary Figure S3). We therefore next examined how these gene-level responses were organized across major metabolic and cellular pathways.

Genes associated with ammonia oxidation, nitrogen metabolism, and cellular stress showed similarly nonuniform responses at pH 5.0. Transcript abundance of *amoA* and *amoB* changed little, whereas two *amoC*-assigned genes were significantly downregulated and two *hao*-assigned genes showed lower transcript abundance without meeting both predefined differential-expression criteria. Genes associated with nitrite and nitric-oxide reduction generally showed modest decreases, whereas several genes involved in urea uptake, hydrolysis, and urease maturation were significantly upregulated (Supplementary Table S1). Stress- and homeostasis-associated genes also showed mixed responses: genes associated with Na^+^/H^+^ exchange, stress-responsive sigma factors, membrane-protein quality control, and selected oxidative-stress defenses were significantly upregulated, whereas a *groEL*-assigned chaperonin, an Fe/Mn superoxide dismutase, and another peroxiredoxin were significantly downregulated (Supplementary Table S1). Thus, acidic pH did not induce a uniform generalized stress-response program but instead altered specific nitrogen-metabolism and cellular-homeostasis functions.

### 3.1 Central carbon pathways show distinct transcriptional responses at pH 5.0

Throughout the Results, “higher” and “lower” refer to transcript abundance at pH 5.0 relative to pH 8.0; statistical significance is stated explicitly. Mapping gene-level expression profiles onto central carbon pathways revealed distinct transcriptional patterns at pH 5.0 (Figure 1, Supplementary Table S1). Within the CBB cycle, transcript abundance was lower for the large and small subunits of RuBisCO (K01601 and K01602), while ribose-5-phosphate isomerase A (K01807), phosphoglycerate kinase (K00927), and glyceraldehyde-3-phosphate dehydrogenase (K00134) were significantly downregulated. By contrast, phosphoribulokinase (K00855) showed higher transcript abundance (Figure 1). Thus, the examined CBB-cycle genes did not respond uniformly, although most genes associated with CO_2_ fixation and downstream reduction showed lower transcript abundance at pH 5.0.

**Figure 1:**
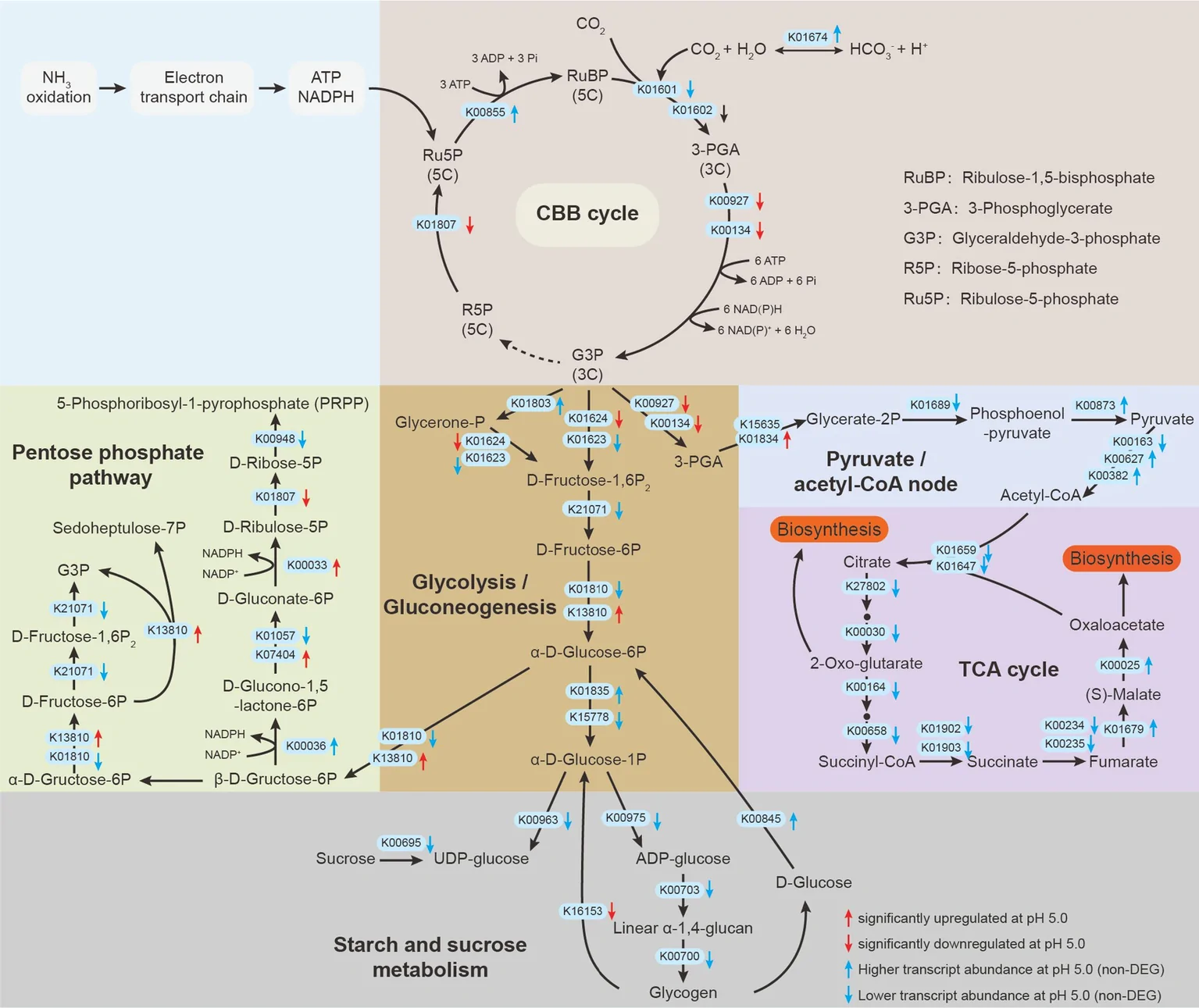
Transcript-abundance differences in central carbon assimilation and biosynthetic metabolism under acidic conditions. The schematic summarizes the metabolic connections among ammonia oxidation, the electron transport chain, the Calvin–Benson–Bassham cycle, the pentose phosphate pathway, glycolysis/gluconeogenesis, the pyruvate–acetyl-CoA node, the tricarboxylic acid cycle, and starch and sucrose metabolism. Blue labels indicate KEGG Orthology (KO) identifiers, and background colors distinguish the major metabolic modules. Black curved arrows indicate metabolic or functional connections. Red upward and downward arrows adjacent to KO identifiers indicate significant upregulation and downregulation at pH 5.0 relative to pH 8.0, whereas blue upward and downward arrows indicate higher or lower transcript abundance, respectively, for genes that did not meet both predefined differential-expression criteria.

The pentose phosphate pathway (PPP) showed a different pattern. Transcript abundance was higher for glucose-6-phosphate 1-dehydrogenase (K00036), and 6-phosphogluconolactonase (K07404) and 6-phosphogluconate dehydrogenase (K00033) were significantly upregulated. The alternative 6-phosphogluconolactonase K01057 showed lower transcript abundance (Figure 1). PRPP synthetase (K00948), which directs ribose-5-phosphate toward nucleotide-precursor formation, also showed lower transcript abundance. These data indicate higher transcript abundance for selected oxidative PPP genes but not for all genes in the pathway or the PRPP-forming branch.

Genes associated with glycolysis/gluconeogenesis and the pyruvate–acetyl-CoA node also showed divergent responses. Transcript abundance was higher for triosephosphate isomerase (K01803). Fructose-bisphosphate aldolases K01624, phosphoglycerate kinase (K00927), and glyceraldehyde-3-phosphate dehydrogenase (K00134) were significantly downregulated, whereas fructose-bisphosphate aldolase K01623 and the phosphofructokinase-family protein (K21071) showed lower transcript abundance (Figure 1). Within the pyruvate dehydrogenase complex, the E1 component (K00163) showed lower transcript abundance, whereas the E2 component, dihydrolipoamide acetyltransferase (K00627), and the E3 component, dihydrolipoamide dehydrogenase (K00382), showed higher transcript abundance. Thus, the three components differed in the direction of their transcriptional responses.

Transcript abundance was lower for most genes assigned to the oxidative TCA cycle, including citrate synthase (K01647), 2-methylcitrate synthase PrpC (K01659), aconitate hydratase A/2-methylisocitrate dehydratase (K27802), NAD^+^-dependent isocitrate dehydrogenase (K00030), the 2-oxoglutarate dehydrogenase components K00164 and K00658, succinyl-CoA synthetase subunits K01902 and K01903, and succinate dehydrogenase subunits K00234 and K00235 (Figure 1). By contrast, fumarate hydratase (K01679) and malate dehydrogenase (K00025) showed higher transcript abundance.

Transcripts assigned to K00963, K00975, K00703, and K00700 showed lower abundance at pH 5.0, whereas the putative glycogen phosphorylase/synthase K16153 was significantly downregulated (Figure 1). Across central carbon metabolism, lower transcript abundance predominated among genes associated with the CBB cycle, the oxidative TCA cycle, and glycogen metabolism, whereas selected oxidative PPP genes showed higher abundance.

### 3.2 Nucleotide and DNA-precursor biosynthesis show predominantly lower transcript abundance at pH 5.0

Genes involved in nucleotide-precursor synthesis generally showed lower transcript abundance at pH 5.0 (Figure 2; Supplementary Table S1). PRPP synthetase (K00948) showed lower transcript abundance, as did most genes assigned to *de novo* purine synthesis from PRPP to IMP, including K01589, K01933, K01756, K01945, K01952, K01923, K00602, and K01588 (Figure 2). Amidophosphoribosyltransferase (K00764) showed higher transcript abundance, but the PRPP-to-IMP branch was dominated by genes with lower abundance.

**Figure 2:**
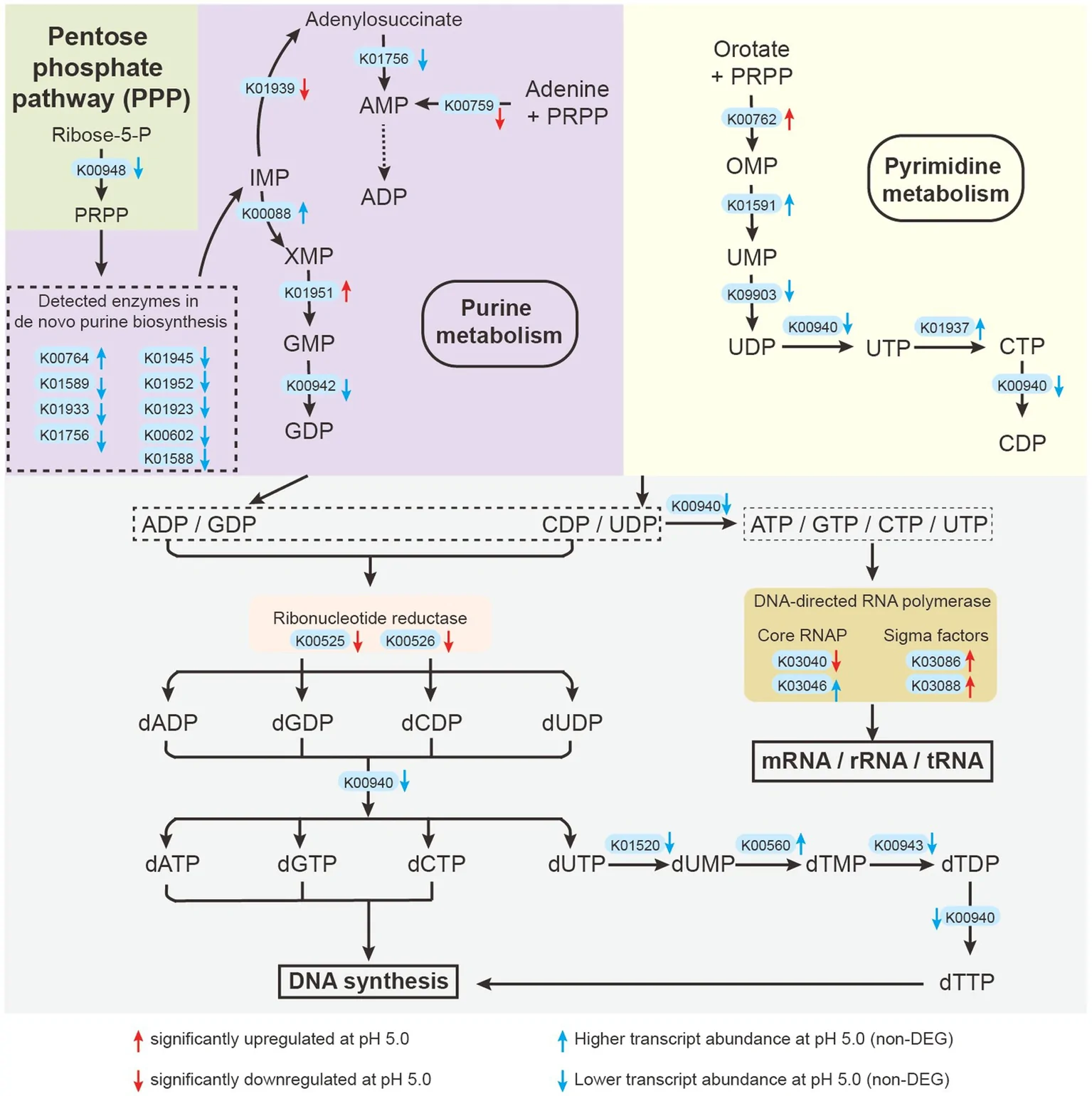
Transcript-abundance differences in genes associated with nucleotide metabolism and nucleic acid synthesis under acidic conditions. The schematic summarizes the connections among the pentose phosphate pathway, purine and pyrimidine metabolism, ribonucleotide-to-deoxyribonucleotide conversion, and DNA and RNA synthesis. Blue labels indicate KEGG Orthology (KO) identifiers, and background colors distinguish the major metabolic and biosynthetic modules. Black curved arrows indicate metabolic or functional connections. Red upward and downward arrows adjacent to KO identifiers indicate significant upregulation and downregulation at pH 5.0 relative to pH 8.0, whereas blue upward and downward arrows indicate higher or lower transcript abundance, respectively, for genes that did not meet both predefined differential-expression criteria.

The AMP branch showed a similar pattern. Adenylosuccinate synthase (K01939) and adenine phosphoribosyltransferase (K00759) were significantly downregulated, whereas adenylosuccinate lyase (K01756) showed lower transcript abundance (Figure 2). The GMP branch showed mixed responses: IMP dehydrogenase (K00088) had higher transcript abundance, GMP synthase (K01951) was significantly upregulated, and guanylate kinase (K00942) had lower abundance. Pyrimidine-metabolism genes were also heterogeneous. Orotate phosphoribosyltransferase (K00762) was significantly upregulated, and orotidine-5′-phosphate decarboxylase (K01591) and CTP synthase (K01937) showed higher transcript abundance, whereas UMP kinase (K09903) and nucleoside-diphosphate kinase (K00940) showed lower abundance (Figure 2).

Genes supporting deoxyribonucleotide synthesis showed a clearer downward pattern. The alpha and beta subunits of ribonucleoside-diphosphate reductase (K00525 and K00526) were both significantly downregulated, and nucleoside-diphosphate kinase (K00940), which contributes to dNDP-to-dNTP conversion, showed lower transcript abundance (Figure 2). Within the dTTP branch, dUTP diphosphatase (K01520) and dTMP kinase (K00943) had lower transcript abundance, whereas thymidylate synthase (K00560) had higher abundance. The higher abundance of thymidylate synthase did not extend across the complete dTTP branch, and the overall dNTP-synthesis profile was dominated by genes with lower transcript abundance.

Components of the transcription machinery also responded differently. The RNA polymerase alpha subunit RpoA (K03040) was significantly downregulated, whereas the beta-prime subunit RpoC (K03046) showed higher transcript abundance (Figure 2). The primary sigma factor RpoD (K03086) and the ECF-family sigma factor RpoE (K03088) were significantly upregulated. These results indicate component-specific changes rather than a consistent directional response across the transcription machinery.

### 3.3 Cell-envelope maintenance and septal peptidoglycan synthesis show contrasting expression patterns

Cell-envelope genes differed between pathways associated with envelope modification and those associated with septal synthesis (Figure 3; Supplementary Table S1). In the ADP-heptose/LPS-core pathway, transcript abundance was higher for the bifunctional HldE (K03272), while D-glycero-D-manno-heptose-1,7-bisphosphate phosphatase GmhB (K03273) and the LPS-core heptosyltransferases WaaF (K02843) and WaaQ (K02849) were significantly upregulated (Figure 3). Thus, multiple genes involved in LPS inner-core assembly showed higher transcript abundance at pH 5.0.

**Figure 3:**
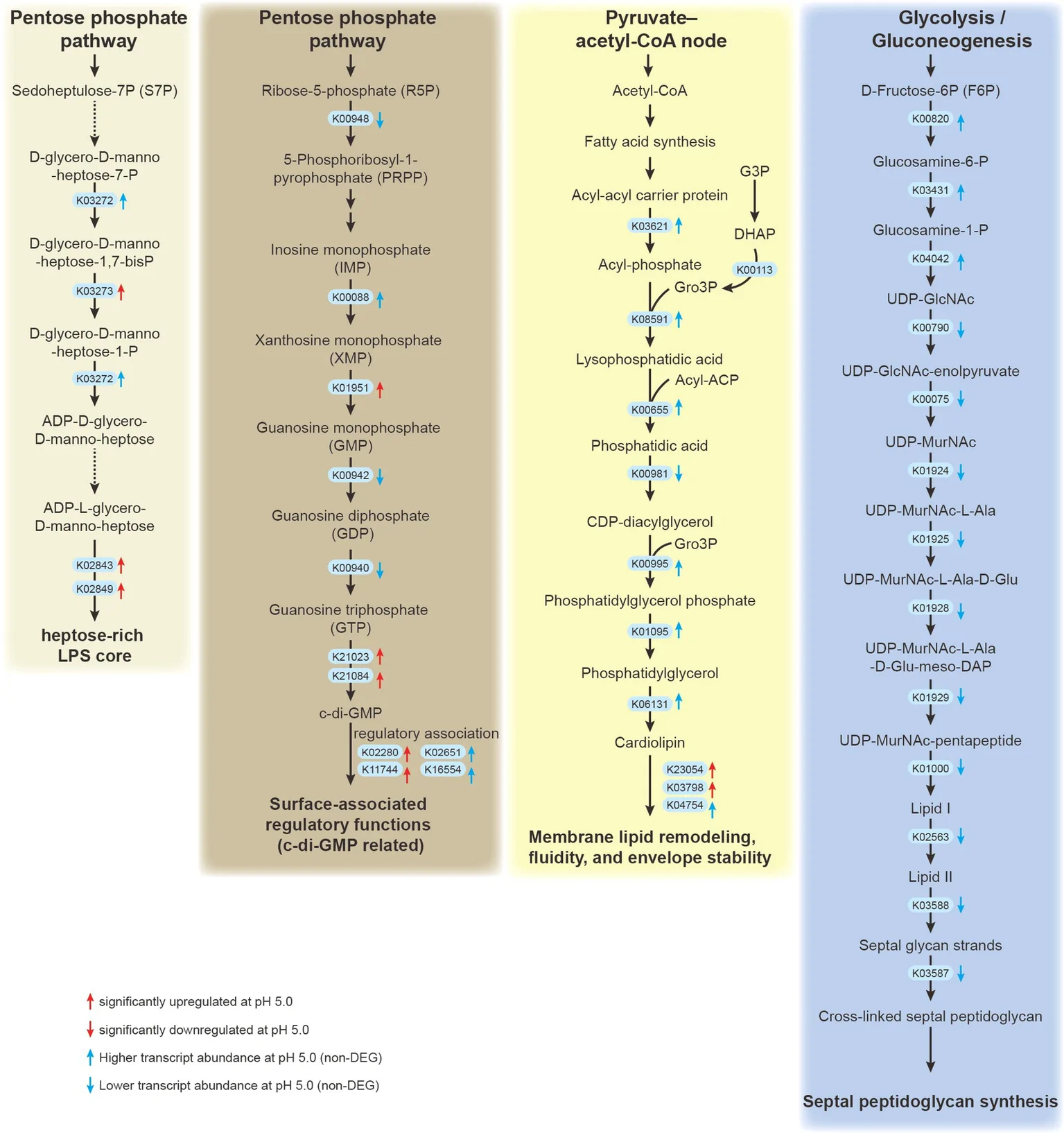
Transcript-abundance differences in genes associated with cell-surface functions, membrane remodeling, and cell-envelope biosynthesis under acidic conditions. The schematic summarizes the links between central carbon metabolism and the biosynthesis of the lipopolysaccharide core, c-di-GMP, membrane lipids, and septal peptidoglycan. Blue labels indicate KEGG Orthology (KO) identifiers, and background colors distinguish the major metabolic and cell-envelope modules. Black curved arrows indicate metabolic or functional connections. Red upward and downward arrows adjacent to KO identifiers indicate significant upregulation and downregulation at pH 5.0 relative to pH 8.0, whereas blue upward and downward arrows indicate higher or lower transcript abundance, respectively, for genes that did not meet both predefined differential-expression criteria. Dashed black connections indicate pathway steps for which the corresponding enzymes were not detected.

Two putative diguanylate cyclases (K21023 and K21084) were significantly upregulated (Figure 3). Among the surface-associated genes, pilus assembly protein CpaC (K02280) and AI-2 transport protein TqsA (K11744) were significantly upregulated, whereas Flp/PilA (K02651) and the putative polysaccharide-biosynthesis transport protein/bacterial tyrosine kinase K16554 showed higher transcript abundance. Together, these genes showed a coordinated pattern of higher transcript abundance, although the corresponding effects on cell-surface phenotypes were not assessed.

Membrane-lipid-related genes showed mixed transcriptional responses. Transcript abundance was higher for PlsX (K03621), PlsY (K08591), PlsC (K00655), PgsA (K00995), PgpA (K01095), and cardiolipin synthase (K06131), whereas CdsA (K00981) showed lower abundance (Figure 3). The putative sn-1 stearoyl-lipid 9-desaturase DesC (K23054) and the membrane-associated AAA protease FtsH (K03798) were significantly upregulated, and MlaA (K04754), which is involved in outer-membrane phospholipid homeostasis, showed higher transcript abundance. No single directional response therefore characterized the complete membrane-lipid pathway.

By contrast, most genes involved in septal peptidoglycan synthesis showed lower transcript abundance (Figure 3). The early UDP-GlcNAc-forming genes *glmS* (K00820), *glmM* (K03431), and *glmU* (K04042) showed higher abundance, whereas the downstream peptidoglycan-specific genes *murA* (K00790), *murB* (K00075), *murC* (K01924), *murD* (K01925), *murE* (K01928), and *murF* (K01929) showed lower abundance. *mraY* (K01000) and *murG* (K02563), which are required for Lipid I and Lipid II formation, were also lower, as were the septal peptidoglycan glycosyltransferase *ftsW* (K03588) and the transpeptidase *ftsI*/*PBP3* (K03587). Thus, higher transcript abundance of early UDP-GlcNAc-synthesis genes contrasted with lower abundance of downstream genes involved in septal peptidoglycan synthesis.

### 3.4 Iron acquisition and cofactor pathways are selectively reorganized at pH 5.0

Genes associated with iron acquisition and iron-dependent cofactors showed different transcriptional directions at pH 5.0 (Figure 4; Supplementary Table S1). The catecholate siderophore receptor K16090 showed higher transcript abundance, and the iron-complex outer-membrane receptor K02014 was significantly upregulated. By contrast, components assigned to two Fe-associated ABC transporter families generally showed lower transcript abundance, including the Fe^3+^ transport ATP-binding protein K02010 and permease K02011, as well as the iron-complex transport ATP-binding protein K02013 and permease K02015. The Mn^2+^/Fe^2+^ transport system showed a different pattern: the substrate-binding protein K09818 was significantly upregulated, and the permease K09819 and ATP-binding protein K09820 showed higher transcript abundance. TonB (K03832) was significantly upregulated and ExbB (K03561) showed higher abundance, whereas ExbD (K03559) showed lower abundance. These patterns differed among transporter systems and did not indicate uniform induction of iron uptake.

**Figure 4:**
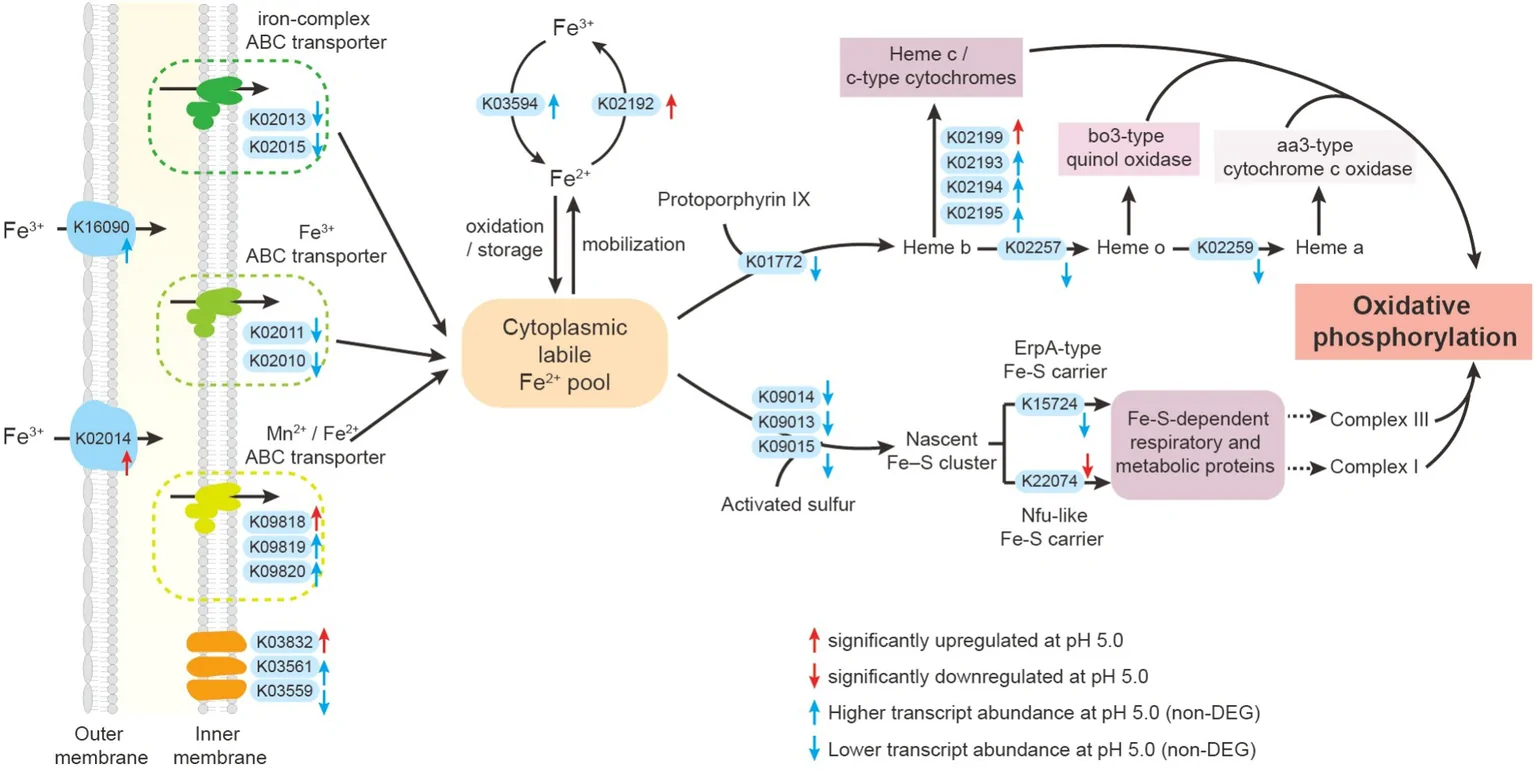
Transcript-abundance differences in genes associated with iron acquisition, iron-cofactor biogenesis, and respiratory cofactor pathways under acidic conditions. The schematic summarizes the connections among transmembrane iron transport, cytoplasmic iron homeostasis, heme and iron–sulfur (Fe–S) cluster biogenesis, respiratory-chain components, and oxidative phosphorylation. Blue labels indicate KEGG Orthology (KO) identifiers, and green dashed boxes denote ABC transport systems. Black curved arrows indicate metabolic or functional connections. Red upward and downward arrows adjacent to KO identifiers indicate significant upregulation and downregulation at pH 5.0 relative to pH 8.0, whereas blue upward and downward arrows indicate higher or lower transcript abundance, respectively, for genes that did not meet both predefined differential-expression criteria. Dashed black connections indicate pathway steps for which the corresponding enzymes were not detected.

Genes associated with iron storage and mobilization also differed. Transcript abundance was higher for bacterioferritin (Bfr, K03594), and bacterioferritin-associated ferredoxin (Bfd, K02192) was significantly upregulated (Figure 4).

Genes involved in heme biosynthesis and cofactor maturation showed contrasting responses. Transcript abundance was lower for ferrochelatase HemH (K01772), heme o synthase CtaB/CyoE (K02257), and heme a synthase CtaA (K02259) (Figure 4). By contrast, the c-type cytochrome maturation component CcmG/DsbE (K02199) was significantly upregulated, and CcmA (K02193), CcmB (K02194), and CcmC (K02195) showed higher transcript abundance. Genes involved in Fe–S cluster assembly generally showed lower abundance, including *sufB* (K09014), *sufC* (K09013), and *sufD* (K09015). The ErpA-type Fe–S cluster insertion protein (K15724) also showed lower transcript abundance, and the putative Nfu-like Fe–S cluster scaffold/carrier protein assigned to K22074 was significantly downregulated.

Overall, the transcriptional response of iron-related pathways was heterogeneous. Selected metal-uptake components, the Bfr–Bfd system, and c-type cytochrome maturation genes showed higher transcript abundance, whereas genes involved in heme a/o synthesis, SUF-mediated Fe–S cluster assembly, and downstream Fe–S cluster transfer showed lower abundance.

## 4 Discussion

Comparative pathway mapping revealed a coordinated but nonuniform transcriptional reorganization in *Nitrosospira* sp. NT at pH 5.0. Lower transcript abundance predominated among genes involved in carbon assimilation, nucleotide and deoxyribonucleotide biosynthesis, glycogen metabolism, and septal peptidoglycan synthesis (Figures 1–3). By contrast, several genes linked to redox support, cell-envelope homeostasis, membrane-protein quality control, surface-associated regulation, and intracellular iron reutilization were not reduced or showed higher abundance (Figures 1, 3, 4). Importantly, the response was not adequately described by a simple global shift from “growth” to “maintenance,” because individual pathways within these broad functional categories also differed substantially. For example, selected oxidative pentose phosphate pathway reactions were relatively preserved while other central-carbon pathways were lower, and intracellular iron mobilization and c-type cytochrome maturation differed from heme a/o synthesis and SUF-mediated Fe–S cluster assembly. These coordinated but nonuniform pathway-level responses are summarized in Figure 5.

**Figure 5:**
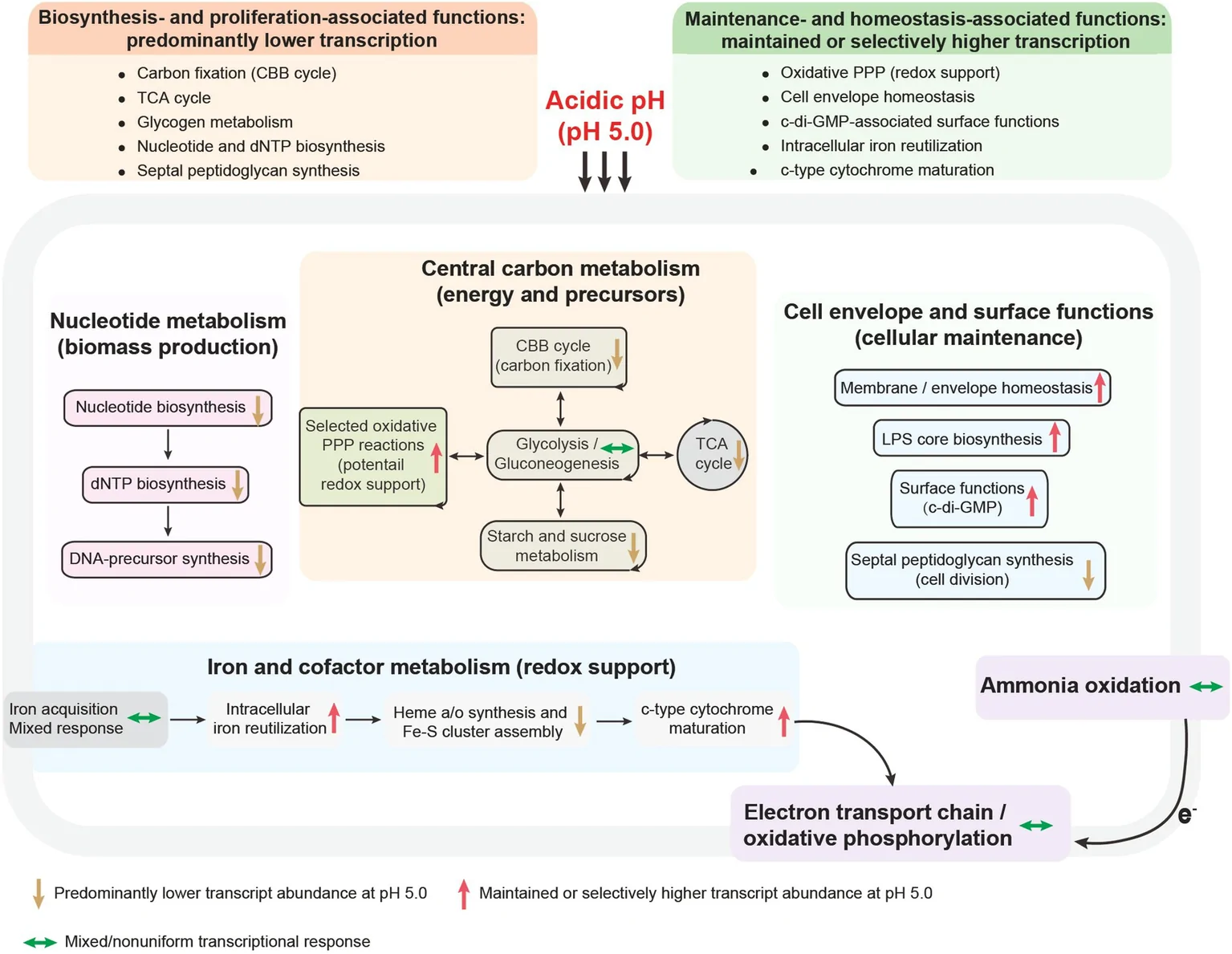
Integrative model of the pathway-level transcriptional response of *Nitrosospira* sp. NT to acidic pH. The schematic summarizes the major transcriptional patterns observed at pH 5.0 relative to pH 8.0 across pathways associated with biomass production, cellular maintenance and homeostasis, redox and cofactor metabolism, ammonia oxidation, and energy metabolism. Brown downward arrows indicate pathways showing predominantly lower transcript abundance at pH 5.0, red upward arrows indicate functions that were relatively maintained or showed selectively higher transcript abundance, and green bidirectional arrows indicate mixed or nonuniform transcriptional responses. The model represents a pathway-level synthesis of the transcriptomic data and does not imply direct changes in metabolic flux, enzyme activity, or cellular resource allocation.

This selective pattern extended to ammonia-oxidation and stress-associated functions. Transcript abundance of *amoA* and *amoB* changed little, whereas two *amoC*-assigned genes were significantly downregulated and two *hao*-assigned genes showed lower transcript abundance without meeting both predefined differential-expression criteria. Thus, the ammonia-oxidation machinery did not respond as a uniformly regulated transcriptional module. Stress- and homeostasis-associated functions were similarly heterogeneous: genes associated with Na^+^/H^+^ exchange, stress-responsive sigma factors, membrane-protein quality control, and selected oxidative-stress defenses were upregulated, whereas other functions, including a *groEL*-assigned chaperonin, an Fe/Mn superoxide dismutase, and another peroxiredoxin, were downregulated. These contrasting responses further argue against a uniform generalized stress program at pH 5.0 and instead support selective transcriptional adjustment of specific cellular functions.

Taken together, the biological insight of these results is not simply that unfavorable acidic conditions were associated with lower expression of growth-related functions. Rather, the data identify which cellular processes were selectively reduced, preserved, or increased and reveal that functionally related maintenance and cofactor pathways were themselves differentially regulated. This organization provides a more specific transcriptional framework for understanding how an acid-tolerant AOB may maintain cellular integrity and essential functions when conditions constrain biomass production. Because transcript abundance does not directly demonstrate metabolic flux, physiological activity, or cellular resource allocation, we interpret this pattern as a pathway-level transcriptional model rather than direct evidence of energetic reprioritization. The following sections examine this selective reorganization in carbon and nucleotide metabolism, cell-envelope homeostasis, and iron-dependent cofactor pathways.

### 4.1 Lower expression of pathways supporting biomass production and proliferation

Several components of central carbon metabolism supported this interpretation. Transcript abundance was lower for RuBisCO subunits and genes involved in the reduction of fixed carbon to triose phosphates (Figure 1), consistent with observations that energetic or substrate limitation can restrict carbon fixation and biomass yield in AOB (Zorz et al., 2018). Although phosphoribulokinase showed higher transcript abundance, this isolated response does not indicate greater CBB-cycle activity because several other genes in the pathway were lower. The data therefore indicate reduced expression of major carbon-assimilation components rather than coordinated activation of the complete CBB cycle (Stein et al., 2013; Zorz et al., 2018).

The TCA cycle and glycogen metabolism showed a related but nonuniform pattern. Transcript abundance was lower for most genes assigned to the oxidative TCA cycle, whereas genes around the malate–oxaloacetate node showed higher abundance (Figure 1). This profile may reflect selective transcriptional maintenance of individual TCA-cycle reactions potentially associated with anaplerotic or precursor-generating functions rather than coordinated activation of the complete oxidative cycle, although metabolic-flux measurements would be required to test this interpretation (Hommes et al., 2006; Norton et al., 2008; Deutch, 2013). Lower transcript abundance of genes involved in ADP-glucose formation and glycogen metabolism further suggests reduced expression of carbon-storage pathways under acidic conditions (Norton et al., 2008).

Selected genes in the oxidative pentose phosphate pathway showed higher transcript abundance (Figure 1). Because oxidative PPP reactions can generate NADPH, this response may contribute to reducing-power requirements associated with redox homeostasis and cellular repair (Spaans et al., 2015; Lawson et al., 2021). This interpretation remains tentative because transcript abundance does not directly measure enzyme activity, NADPH production, or pathway flux (Hoppe, 2012; Chubukov et al., 2013). Nevertheless, higher abundance of selected oxidative PPP transcripts together with lower abundance of PRPP synthetase is compatible with greater relative expression of reactions that can support NADPH generation and lower expression of PRPP-dependent biosynthesis, including nucleotide-precursor production (Spaans et al., 2015; Hove-Jensen et al., 2017).

Nucleotide metabolism further supports reduced expression of growth-associated biosynthesis. Most genes assigned to *de novo* purine synthesis showed lower transcript abundance, and both ribonucleotide-reductase subunits were significantly downregulated (Figure 2). Because ribonucleotide reductase converts ribonucleotides to deoxyribonucleotides, this profile is consistent with lower expression of DNA-precursor synthesis (Nordlund and Reichard, 2006). Lower abundance of nucleoside-diphosphate kinase and several dTTP-associated genes supports the same interpretation, although intracellular dNTP concentrations or imbalances cannot be inferred from transcript data alone (Norton et al., 2008; Schaaper and Mathews, 2013). Individual genes in the GMP and CTP branches showed higher abundance, demonstrating that nucleotide metabolism was not uniformly reduced and possibly reflecting continued transcriptional demand for RNA synthesis, guanine-nucleotide signaling, or CTP-dependent membrane biosynthesis. The higher abundance of thymidylate synthase may reflect a continuing requirement for DNA maintenance, but this possibility remains speculative (Hove-Jensen et al., 2017; Pozzi et al., 2019).

The peptidoglycan pathway provides an independent transcriptional indication of lower expression of proliferation-associated functions. Genes involved in Mur-dependent precursor synthesis, Lipid II formation, and FtsW/FtsI-mediated septal polymerization and cross-linking generally showed lower transcript abundance (Figure 3), whereas early UDP-GlcNAc-synthesis genes showed higher abundance. Because UDP-GlcNAc is a shared precursor for peptidoglycan and other cell-envelope pathways, including lipid A–lipopolysaccharide biosynthesis, this divergence suggests that precursor formation may have been maintained while expression of septal synthesis declined (Typas et al., 2011; Egan et al., 2020; Sachla and Helmann, 2021; Hummels et al., 2023). However, no direct measurement of growth or cell division was made; these results therefore indicate lower transcriptional commitment to septal peptidoglycan synthesis rather than demonstrating reduced division. Taken together, the carbon-assimilation, nucleotide, and peptidoglycan profiles support a coordinated reduction in the expression of pathways associated with biomass accumulation and proliferation at pH 5.0.

### 4.2 Cell-envelope maintenance and remodeling under acidic conditions

In contrast to the septal peptidoglycan genes, several genes associated with modification of the existing cell envelope showed higher transcript abundance (Figure 3). Higher abundance of *hldE* and significant upregulation of *gmhB*, *waaF*, and *waaQ* indicate increased expression of genes involved in heptose-containing LPS inner-core biosynthesis (Kneidinger et al., 2002; Cote and Taylor, 2017). If these transcriptional changes alter LPS composition, they could affect outer-membrane architecture, permeability, or interactions with the acidic extracellular environment (Hong et al., 2018). Membrane-phospholipid genes showed mixed directional responses, which is more compatible with selective remodeling than with uniform expansion of membrane biomass (Chang and Cronan, 1999; Zhang and Rock, 2008). Direct lipid and LPS measurements would be required to establish the resulting compositional changes.

Significant upregulation of a putative DesC-like fatty-acid desaturase could affect acyl-chain packing and membrane fluidity through changes in fatty-acid unsaturation (Willdigg and Helmann, 2021). The higher abundance of ftsH transcripts suggests increased expression of membrane-protein quality-control machinery, while higher *mlaA* transcript abundance is compatible with maintenance of outer-membrane phospholipid asymmetry (Mitchell and Silhavy, 2019; Chai-Danino et al., 2026). Together, these genes were among the envelope-maintenance functions preserved or more highly expressed at pH 5.0. Their physiological effects, however, cannot be established without measurements of protein abundance, membrane composition, or envelope integrity.

The two significantly upregulated putative diguanylate cyclase genes and the higher abundance of several surface-associated transcripts indicate altered expression of c-di-GMP-related and cell-surface functions (Figure 3). Because c-di-GMP commonly regulates surface-associated behaviors, these changes suggest modified regulatory potential under acidic conditions (Poulin and Kuperman, 2021; Webster et al., 2021). Surface-associated communities can protect cells from chemical stress by modifying diffusion and creating heterogeneous local microenvironments (Flemming et al., 2016). Nevertheless, the present data do not demonstrate increased attachment, aggregation, microcolony development, or biofilm formation.

More specifically, significant upregulation of the two putative diguanylate cyclase genes does not establish greater enzyme activity, altered intracellular c-di-GMP concentrations, or direct regulation of the co-expressed surface-associated genes. Direct c-di-GMP measurements, enzymatic characterization, and targeted attachment, aggregation, or biofilm assays would be required to test these possibilities (Petchiappan et al., 2020; Richter et al., 2020). The co-occurrence of c-di-GMP-associated responses with flagellar-assembly and chemotaxis transcripts is not necessarily inconsistent because motility and surface-associated behaviors may occur sequentially or in different cellular subpopulations (Armbruster et al., 2019; Colin et al., 2021). We therefore interpret these data as evidence of altered cell-surface regulatory potential rather than a uniform transition from motile to sessile growth.

### 4.3 Reorganization of iron acquisition, storage, and cofactor pathways

Iron metabolism did not show uniform transcriptional induction at pH 5.0. Components of Fe^3+^ and iron-complex ABC transporter families generally showed lower transcript abundance, whereas genes assigned to the Mn^2+^/Fe^2+^ transport system showed higher abundance. The TonB–ExbB–ExbD complex also showed mixed responses, with significant upregulation of *tonB*, higher *exbB* abundance, and lower *exbD* abundance (Figure 4). Low pH therefore affected individual metal-uptake routes differently, and the transcript data do not support generalized enhancement of iron acquisition.

Bacterioferritin *bfr* showed higher transcript abundance, and its putative associated ferredoxin *bfd* was significantly upregulated. Bfd can facilitate mobilization of iron stored in bacterioferritin (Weeratunga et al., 2009; Punchi Hewage et al., 2020); accordingly, this expression pattern is compatible with increased transcriptional potential for intracellular iron mobilization. It does not, however, demonstrate Bfr–Bfd activity, iron flux, or reduced dependence on environmental iron acquisition.

Higher expression of the Bfr–Bfd system was not accompanied by broad induction of iron-dependent cofactor-biosynthesis genes. Transcript abundance was lower for *hemH*, *ctaB*/*cyoE*, and *ctaA*, whereas several components of the Ccm system showed higher abundance, including significant upregulation of *ccmG* (Figure 4). This divergence suggests relative preservation of c-type cytochrome maturation compared with heme a/o biosynthesis, although functional maturation also depends on heme availability (Stevens et al., 2011; Hederstedt, 2012; Dailey et al., 2017). The lower abundance of *sufB*, *sufC*, *sufD*, and Fe–S cluster-carrier genes likewise argues against broad transcriptional stimulation of *de novo* Fe–S cluster assembly and transfer (Ayala-Castro et al., 2008; Chahal et al., 2009). Protein and cofactor measurements are needed to determine the functional consequences of these expression patterns.

The mixed responses of cofactor-related genes constrain interpretation of oxidative phosphorylation. Respiratory-chain genes and the heme- and Fe–S-associated pathways that support their assembly showed both higher and lower transcript abundance; consequently, the data do not indicate global activation of oxidative phosphorylation at pH 5.0 (Shepherd et al., 2010; Stein et al., 2013; Kaila and Wikstrom, 2021). Instead, they indicate differential expression among respiratory components and cofactor-maturation modules. Because transcript abundance does not directly measure protein activity, cofactor occupancy, electron-transfer rates, or metabolic flux, the proposed maintenance-oriented response should be regarded as a pathway-level transcriptional model rather than direct evidence of cellular resource allocation (Hoppe, 2012; Chubukov et al., 2013).

An additional limitation is that the experiments were conducted with an enrichment culture rather than a pure culture of *Nitrosospira* sp. NT. The pH 5.0 and pH 8.0 treatments were established from the same parent enrichment and incubated for 24 h, but treatment-specific DNA-based community profiles were not obtained. Although many of the major transcriptionally represented taxa were detected under both conditions, their relative metatranscriptomic contributions differed, and RNA-based taxonomic profiles cannot distinguish changes in population abundance from changes in transcriptional activity. We therefore cannot exclude the possibility that pH-dependent changes in the activity or interactions of other community members indirectly contributed to the transcriptional response of *Nitrosospira* sp. NT. Future studies using treatment-specific DNA-based community profiling and, ultimately, a pure culture of strain *Nitrosospira* sp. NT will be required to distinguish direct responses to pH from potential community-mediated effects.

Taken together, these pathway-specific responses provide a potential mechanistic context for the persistence of bacterial ammonia oxidizers in acidic environments. Acid-tolerant *Nitrosospira* have been identified as active contributors to nitrification under low-pH conditions, yet acid tolerance does not necessarily imply optimal growth or biomass production. The present results suggest that persistence under such conditions may involve maintaining selected functions related to cellular integrity, redox support, surface regulation, and intracellular cofactor management while showing lower transcript abundance in several pathways associated with biomass accumulation and proliferation. Such persistence may help sustain ammonia-oxidizing potential even when growth-associated functions are constrained and could therefore be relevant to the temporal stability and recovery of nitrification following acidification. At the ecosystem scale, this may be relevant to nitrate production and downstream nitrogen transformations in acidic environments. More broadly, the maintenance-oriented transcriptional pattern identified here may provide mechanistic context for interpreting pH-dependent representations of nitrification in biogeochemical models. However, the present transcriptomic data do not directly quantify nitrification rates, nitrogen fluxes, or model parameters, and therefore cannot by themselves establish ecosystem-scale effects on nitrogen cycling.

Future studies should combine transcriptomic analyses with direct measurements of ammonia-oxidation rates, biomass production, cellular energetic status, protein abundance, metabolite concentrations, and nitrogen-product fluxes across broader pH gradients and time scales. Such measurements would help determine whether the transcriptional patterns identified here correspond to changes in nitrification performance and nitrogen flux. In addition, factorial experiments combining pH with temperature and other climate-related environmental drivers would be valuable for determining whether the maintenance-oriented response identified here persists under changing environmental conditions.

## 5 Conclusion

Genome-resolved metatranscriptomic analysis revealed that the response of *Nitrosospira* sp. NT to pH 5.0 was characterized by selective pathway-level reorganization rather than uniform transcriptional suppression. Genes associated with carbon assimilation, nucleotide and deoxyribonucleotide biosynthesis, glycogen metabolism, and septal peptidoglycan synthesis generally showed lower transcript abundance, whereas selected functions related to redox support, cell-envelope homeostasis, surface-associated regulation, intracellular iron mobilization, and c-type cytochrome maturation were relatively preserved or increased. Importantly, maintenance- and cofactor-related pathways also responded nonuniformly, as illustrated by the contrasting transcriptional patterns among iron mobilization, cytochrome c maturation, heme a/o biosynthesis, and Fe–S cluster assembly. These findings extend the general growth-versus-maintenance concept by identifying the specific cellular processes that are differentially maintained under acidic conditions and provide a pathway-level framework for understanding how acid-tolerant AOB may preserve essential functions and persist as contributors to nitrification when low pH constrains biomass production. Such persistence may be relevant to the stability and recovery of nitrification in acidic environments and may provide mechanistic context for interpreting pH-dependent responses of nitrogen cycling.

## Data Availability

The raw sequence data reported in this paper are available in the National Center for Biotechnology Information Sequence Read Archive repository (BioProject ID: PRJNA1456123).
