# Supplementary material for: Pathway-level analysis suggests a maintenance-oriented transcriptional response of the ammonia-oxidizing bacterium Nitrosospira sp. NT to acidic pH
Source: Front Microbiol. 2026 Oct 8;17:1957381. doi: 10.3389/fmicb.2026.1957381 (PMC13687032; doi:10.3389/fmicb.2026.1957381)
Supplement: Supplementary file 1 [file Supplementary_file_1.DOCX]

***Supplementary Material***

**Supplementary Figures**

**Figure S1. Taxonomic profiles of the parent enrichment culture and the pH-shifted metatranscriptomes.** (A) Relative genomic representation of the major species-level taxa in the parent enrichment culture maintained at pH 6.0 before establishment of the pH treatments. Metagenomic taxonomic counts were obtained from Kraken2 classification and normalized to the total number of taxonomically assigned reads in each biological replicate. (B) Relative transcript representation of major taxa in the metatranscriptomic libraries after 24 h of incubation at pH 5.0 or pH 8.0. Species-level taxonomic counts were obtained from Kraken2 classification of the metatranscriptomic reads and normalized to the total number of taxonomically assigned reads in each library. R1–R3 indicate three independent biological replicates. The displayed taxa represent the major genomically or transcriptionally represented community members, whereas taxa not shown account for the remaining fraction of each sample. Red boxes highlight the *Nitrosospira*-assigned population. Because taxon-specific RNA representation reflects both population abundance and transcriptional activity, the profiles in panel B should not be interpreted as direct measurements of population-level community composition or relative abundance.

**Figure S2. Reproducibility and global transcriptional variation among *Nitrosospira* sp. NT metatranscriptomic samples.** (A) Pairwise Pearson correlation heatmap of the six metatranscriptomic libraries. Correlations were calculated across CDSs using log2-transformed DESeq2-normalized counts [log2(normalized count+1)] after excluding CDSs with zero counts across all six samples. Values within the matrix indicate Pearson correlation coefficients. (B) Principal component analysis (PCA) of the same transformed count matrix. PC1 and PC2 explained 76.8% and 8.3% of the total variance, respectively. Orange circles represent the pH 5.0 treatment and blue squares represent the pH 8.0 treatment. R1–R3 indicate three independent biological replicates.

**Figure S3. MA plot showing genome-wide differential expression of *Nitrosospira* sp. NT between pH 5.0 and pH 8.0.** The x-axis shows the log10-transformed DESeq2 baseMean, representing the mean normalized expression level across samples, and the y-axis shows the log2 fold change in transcript abundance at pH 5.0 relative to pH 8.0. Genes meeting the predefined criteria for significant differential expression (|log2 fold change| ≥1 and Benjamini–Hochberg-adjusted *P* < 0.05) are shown as significantly upregulated (salmon) or significantly downregulated (blue), whereas genes not meeting both criteria are shown in gray.
